# Retrospect of Miasmatic Diseases, Etc.

**Published:** 1854-02

**Authors:** Hiram Nance

**Affiliations:** Lafayette, Ill.


*Retrospect of Miasmatic Diseases for a Few Years past*, by HIRAM NANCE, M. D., of Lafayette, Ill. Read before the Stark County Medical Society.

I HAVE thought that it might prove interesting to devote a few pages to the subject of our diseases for the last six or seven years, as they occurred also during the past summer and present fall; especially the cases that came immediately under my observation.

It is true, though remarkable, that our diseases in the Western country, so far as my knowledge extends, differ in type every successive year, especially those occurring in the summer and fall, or in other words those of miasmatic origin. I have resided in Stark county for the last seven years, and during that time each successive year has brought something new to the pathologist; why this diversity? I am unable to answer; it seems strange that it should be so when we consider that our country is not peculiarly subject to great vicissitudes during the summer and fall months I have not given meteroology the attention it deserves, in ascertaining the mean temperature for any two or more years, neither have I measured the water that has fallen, nor the bright and beautiful days, or counted the gloomy and cloudy ones. Should this subject be watched as it deserves, probably something might be elicited in making this subject more clear to us. During the summer and fall of 1845, my first year in this part of the State, Intermittents prevailed extensively. They usually prevailed of a pure, undecided character, the cold stage was well marked, characterized frequently with well marked rigors. When this subsided, the fever came on actively, the pulse was full and strong, tongue heavily coated, epigastric tendency well marked, great thirst, and almost constant headache. This variety of disease prevailed principally during the early part of the season, but cases of this description prevailed occasionally, until the latter parts of November; as they gradually subsided, their places were supplied by the best marked cases of Remittent Fever I ever witnessed. The patients some times would be taken down without any premonitory symptoms. First a chill, and then succeeded by fever. At other times, for a day or two or more, the patient would complain of the usual symptoms that precede the disease under consideration, viz.: aching of the head and bones, slight creeps up the back, loss of appetite, bitter taste in the mouth, usually constipated state of the bowels, and a general feeling of *malaise.* After a part, or all of the foregoing symptoms, the fever came on usually about 9 or 10 o'clock A. M., and continued actively until from 2 to 4 o'clock in the morning, then a distinct remission made its appearance, accompanied sometimes with quite a moist state of the surface, and occasionally the breast and upper extremities were in a pretty free state of perspiration, but always in these cases the pulse remained excited, thirst continued, with headache and other well marked febrile symptoms. But rarely would this abatement or remission remain for more than 8 or 10 hours.

Cases of the description that I have just related, were treated by Prot. Chlo. Hydrarg. combined usually with Pulv. Rhei given as a mild cathartic, or the calomel given and followed in from 4 to 5 hours by seidlitz powder, sul. magnesia or castor oil. When the bowels had been properly evacuated, my patient was placed on nitrous or dover powder, combined with i. or ii. grs. of calomel given every three or four hours, until it was thought prudent to move the bowels ; this I attended to at least once a day. If the remission was sufficient, I gave quinia, but rarely did it seem to accomplish much ; and here I would remark, that I have but little confidence in quinia, except in cases where there is almost an entire intermission. Under this treatment, with proper variations, the disease usually gave way from the fourth to the tenth day.

The intermittents were managed easily, a dose of Cook's pills or Cal. and Rhei, followed by 10 or 15 grs.quinia in divided doses usually put a quietus on them. Relapses were not frequent, and when they did occur, sul. quinia was given which, when properly used for fourteen or twenty-one days, the disease but rarely returned. During this season, I saw four or five cases of Congestive Fever, two in my practice died, and two others I thought would, but by the mammoth doses of quinia they were evidently rescued from the jaws of death. Calomel in Congestive Fever of miasmatic origin, is a great medicine, but when compared with the sul. of quinia, it sinks almost into oblivion. In congestive fever, if there is much congestion, I would give quinia in the cold or hot stage, for upon this depends the salvation of our patient.

The year succeeding, which was 1846, was the year known by all who then resided here, as the *sickly year*, and truly it merits this epithet. None of us will ever have it effaced from our memories, neither will those who were then afflicted. The diseases this year were nearly all intermittent, but not well marked, the chill rarely amounted to anything scarcely, sometimes a very slight rigor, at other times coldness of the feet, and occasionally our patients would tell us they had no chilly sensations, yet there would be fever and a distinct *intermission.* These were treated pretty much as the former year. In the month of October, some cases of continued or typhoid fever came on, but they were all mild and gave way under a simple treatment. The next year typhoid came on malignantly, it not only prevailed in Stark county, but it became a general disease of the Western country. This disease had not prevailed extensively (I believe,) in this part of the State at any previous time; though I am informed several deaths occurred from it four or five years before in the prairie south of Lafayette, and some near Wethersfield, in Henry county. Typhoid fever was difficult to manage this year; and the next year, 1848, many cases died, not only in Stark, but in the adjoining counties of Knox, Peoria, etc. I believe Henry country pretty much escaped these two years. It has existed amongst us ever since, but has not assumed such a malignant type. In '50, also in '51, the type has grown milder, or at least is more successfully managed.

Since '49 there has been quite an important change in the character of our summer and fall diseases; instead of intermittent and remittent fevers, their places have been supplied to a very great extent by diseases, whose pathology is gastro-enteric. Last year dysentery prevailed extensively, not only in my immediate vicinity, and in our county, but throughout a great portion of the Western country. There are but few that differ at all on its pathology, but when we come to its treatment, I find quite a diversity of opinion. Some treat it as a phlegmasia, others rely pretty much entirely upon the salts of morphia or pulv. opii. I take the view that there is portal obstruction, and found my treatment upon this theory, combining my alterative with sul. morphia, and acetat. plumbi, with mild laxatives of pulv. rhei or oleum ricini once a day, or at least once every other day. Under this treatment, with warm fomentations, I have had no cause to reflect upon my past practice, or wish I had adopted a different one.

During the present summer and fall, gastro-enteric diseases have also prevailed in a peculiar form. They have not been simple, but in almost every case they are combined with intermittent fever; consequently our treatment has been of a double character, in order to suit our cases.

Amongst children there are but few that have escaped some derangement of the stomach and bowels, especially those under two years of age. It seemed to matter not whether they were cutting teeth or not. Children usually suffered from one day to one or two weeks with diarrhœa, before I had an opportunity of seeing them, then most usually vomiting had made its appearance, constituting cholera infantum; with this vomiting and purging in almost every case chill and fever were connected. Here was a catalogue of symptoms presenting themselves that required some thought and judgment to adopt a proper pathological treatment.

The stomach was irritable, and of course the diarrhœa could not be arrested until medicine could be borne by the stomach; neither could quinine be taken to arrest the chills; for as soon as taken, the stomach would reject it. My treatment was premised by ¼ or ½ gr. calomel, with a few drops ess. peppermint and loaf sugar, given every half hour, or oftener, if vomited up, until the stomach became quiet. If there was not sufficient calomel taken to move the bowels and change the nature of the stools, I then gave a dose of cal. rhei and magnesia, or withheld the former and gave the latter two. This usually produced an improvement in the state of the stomach and bowels. I then gave quinia, and if diarrhœa continued, my patient was placed on a combination of cal. ipecæ. creta, prep. and acetat. plumbi. This treatment usually carried them through successfully.

In adults the disease differed some: usually the vomiting was absent, but in some cases we found it also present. In nearly all cases that came under my care, I found a miasmatic or intermittent state of the system connected with a diarrhœa, either of the serous or bilious variety. The first case I saw, was a lady of some 35 years, it made its appearance as an ordinary intermittent, but bilious diarrhœa soon came on. The case puzzled and perplexed me very much. I gave quinine, preceded by an alterative for the fever, but this wouldn't cure diarrhœa, then I would give medicine for the latter, and the chills would return: at this time I found my patient's stomach so irritable, that it was almost impossible for her to retain a febrifuge; I resolved then to stop giving medicine, except something of a mucilaginous character, this I continued for a day or two, and at this period my patient's stomach grew so quiet, that small quantities of medicine could be borne. Turpentine, acetat. plumbi, and all the usual vegetable astringents were rejected even in the smallest doses. I combatted this diarrhœa by small doses of *hydr. cum creta*, and mucilage of gum acacia, with an epispastic applied on the epigastric region; an occasional opiate enema was administered to quiet pain and produce sleep.

Ordinary cases of diarrhœa and intermittent fever, I treated with a powder composed of cal. grs. i. or ii., sul. morphia grs. ¼ to

½, and acetat. plumbi grs. 3 to 5; at the same time my patient was under treatment for diarrhœa I gave quinine sometimes, instead of the above powder; if the system was not bilious, I gave spts. turpentine and laudanum. When the diarrhœa was controlled, I thought it prudent not to move the bowels for three or four days, unless pain or febrile symptoms made their appearance.

